# Predictive ability of both the healthy aging index and the frailty index for all-cause mortality

**DOI:** 10.1007/s11357-024-01097-0

**Published:** 2024-02-22

**Authors:** Felipe Diaz-Toro, Gabriela Nazar, Alejandra-Ximena Araya, Fanny Petermann-Rocha

**Affiliations:** 1 Facultad de Enfermería, Escuela de Enfermería, Universidad Andrés Bello, Santiago, Chile; 2 Millennium Institute for Care Research (MICARE), Santiago, Chile; 3 Department of Psychology, Universidad de Concepción, Casilla, 160-C Concepción, Chile; 4 School of Cardiovascular and Metabolic Health, University of Glasgow, Glasgow, UK; 5 Centro de Investigación Biomédica, Facultad de Medicina, Universidad Diego Portales, Santiago, Chile

**Keywords:** Frail, Mortality, Comorbidities, Aging

## Abstract

**Aim:**

We aimed to develop and assess a modified healthy aging index (HAI) among Chileans aged 60 years and older and compare its predictive ability for all-cause mortality risk with the frailty index (FI).

**Methods:**

This prospective study analyzed data from the Chilean National Health Survey (CNHS) conducted in 2009–2010. We included 847 adults with complete data to construct the HAI and FI. The HAI comprised five indicators (lung function, systolic blood pressure, fasting glucose, cognitive status, and glomerular filtration rate), while the FI assessed frailty using a 36-item scale. HAI scores were calculated by summing the indicator scores, ranging from 0 to 10, with higher scores indicating poorer health. Receiver operating curves (ROC) and area under the curve (AUC) were used to assess predictive validity. Associations with all-cause mortality were assessed using Cox proportional hazard models adjusted by confounders.

**Results:**

The mean HAI score was 4.06, while the FI score was 0.24. The AUC for mortality was higher for the HAI than the FI (0.640, 95% confidence interval (CI) 0.601 to 0.679 vs. 0.586, 95% CI 0.545 to 0.627). After adjusting for confounders, the FI showed a higher mortality risk compared to the HAI (2.63, 95% CI 1.76 to 3.51 vs. 1.16, 95% CI 1.08 to 1.26).

**Conclusion:**

The FI and HAI were valid predictors for all-cause mortality in the Chilean population. Integrating these indices into research and clinical practice can significantly enhance our capacity to identify at-risk individuals.

**Supplementary Information:**

The online version contains supplementary material available at 10.1007/s11357-024-01097-0.

## Background

With the global increase in the older population, there has been significant attention on preventing or reducing the complications associated with aging [1]. Several indices have been created to evaluate the overall health of older individuals, which measure comorbidities [2], health status and functionality[3], and frailty [4]. These measures have proven to be valuable tools for predicting a wide range of adverse outcomes such as hospitalization, disability, cognitive decline, and mortality [5–7]. Some of these indices are based on recognizable diseases, signs, and symptoms, while others focus on detecting subclinical diseases based on clinical and biological parameters.

Two examples of these different approaches are the frailty index [8] (FI) and the healthy aging index [9] (HAI). The FI states that frailty is caused by the accumulation of health deficits during the life course and that the more deficits a person has, the more likely this person is to be frail [8]. The FI includes symptoms, signs, diseases, and disabilities from different domains (functional, cognitive, and social characteristics) [8] as deficits. On the other hand, the HAI employs surrogate tests such as systolic blood pressure, lung function or forced vital capacity, cystatin C or creatinine, fasting glucose, and Digit Symbol Substitution Test or the Mini-Mental State Examination (MMSE) to assess overall health and predict adverse outcomes in older individuals [9]. Previous research has shown that the HAI is a reliable predictor of mortality. For instance, Wu et al. [10] linked a modified HAI (including various health indicators) to increased all-cause and cardiovascular mortality in older Americans. Another study by Huang et al. [11], using a modified HAI version, found that it was associated with a higher risk of major cardiovascular events. Although the FI and HAI have been shown to be confident predictors of mortality [12, 13], no studies have yet compared their ability to predict mortality in the Chilean population.

The purpose of this study is twofold. Firstly, we aimed to develop and assess a modified version of the HAI in a large, nationally representative sample of Chileans aged 60 years and older. Secondly, we aimed to compare the predictive ability for all-cause mortality of this modified HAI with that of the FI, another commonly used predictor of mortality previously validated in this population. The novelty of our study lies in the utilization of a modified version of the HAI, specifically tailored for the Chilean population, providing a unique perspective on predictive tools for mortality in this demographic.

## Methods

### Study design

This prospective study used data from the Chilean National Health Survey (CNHS) conducted between 2009 and 2010 [14]. The CNHS 2009–2010 is one of Chile’s largest, nationally representative population-based surveys of health conditions, lifestyle, health risk factors, and morbidity in a stratified multistage probability sample of 5416 participants. For the purposes of this research, from 1042 participants aged 60 and older, 847 (81.3%) with complete data to construct the HAI, the FI, and covariates were included in the analyses. No statistical differences were observed between excluded and included participants regarding age (70.1 vs. 70.4, *p* = 0.357) and sex (females 60.1% vs. 63.2%, *p* = 0.178). Prior to participation, all participants provided written consent. The CNHS 2009–2010 was funded by the Chilean Ministry of Health and approved by the Ethics Research Committee of the School of Medicine at the Pontificia Universidad Católica de Chile [14].

### Assessment of the modified healthy aging index

This modified version of the HAI is based on a study by Sanders et al. [13]. The revised HAI consists of five parameters that are classified as 0 (healthy), 1 (intermediate), or 2 (less healthy). The HAI is determined by adding up the scores of all five indicators, resulting in a final index ranging from 0 to 10, where a score of 10 indicates the least healthy state. The cut-off values for these indicators are presented in Supplementary information 1. Our modified version of the HAI is built upon the five parameters proposed by Sanders et al., adapting them to the data available in our dataset. The five indicators included are lung function (measured by self-reported symptoms of expectoration, cough, sibilance, and difficulty in breathing), systolic blood pressure, fasting glucose, cognitive status (assessed through the abbreviated version of the MMSE [15]), and glomerular filtration rate (this was calculated using the plasma value of creatinine through the Cockcroft formula proposed in the CNHS). Tertiles were also created from the continuous HAI, based on the final score: (i) 0 to 3 points (healthier), (ii) 4 to 6 points (intermediate), and (iii) 7 to 10 (less healthy) points. The latter enabled us to meticulously explore the relationship of the index, specifically within the Chilean population. Moreover, this adjustment aligns us for a more meaningful comparison with the frailty index, (which itself is categorized into three distinct groups.) 

### Assessment of the FI

A 36-item FI published and validated elsewhere [7] was used to assess frailty. This FI is based on self-reported data following standard procedures described by Searle et al. [4]. Briefly, to be considered a deficit, a variable must satisfy the following criteria: (i) their prevalence should increase with age; (ii) be associated with health status; and (iii) not saturate too early or have a very low prevalence [4]. All deficits were scored between 0 and 1, where 0 indicates the absence of the deficit and 1 the presence of the deficit. A final frailty score was calculated for each participant by dividing the sum of the health deficit (36-item) scores by the total number of health deficits assessed. Additionally, three categories of the FI were created: (i) < 0.12 points, robust; (ii) > 0.12 to 0.24 points, pre-frail; and (iii) > 0.24 frail.

### All-cause mortality

Data on all-cause mortality, including death dates, were obtained for long-term follow-up by linking the CNHS to the Chilean Civil Registry and Identification. The available mortality data covered a period up to December 31, 2020. Therefore, mortality was either censored on that date or on the actual date of death.

### Covariates

Self-reported data for sociodemographic characteristics, including age, sex, years of education, place of residency, smoking status, and alcohol consumption, were collected from all participants using questionnaires previously validated for the CNHS 2009–10. The following categories were derived for the sociodemographic variables: age (≥ 60), sex (men and women), years of education (≤ 8 years, 9–12 years, and > 12 years), place of residence (urban and rural), and smoking status (never, previous, and current). Alcohol consumption was derived using the Alcohol Use Disorders Identification Test (AUDIT) [16] and categorized as low, moderate, and high risk.

### Statistical analyses

We presented the comparison between the HAI and FI among the participants as mean and standard deviation (SD) for continuous variables and percentages with their 95% confidence intervals (CI) for categorical variables. To assess the predictive validity of the instruments for all-cause mortality, we created receiver operating characteristics (ROC) curves and calculated the area under the curve (AUC) for each of them. Then, we calculated the index’s prevalence, sensitivity, and specificity using the cut-offs previously described.

Cox proportional regression models were used to explore the relationship between HAI and FI and all-cause mortality. Additionally, to compare the predictive ability of mortality, a Harrell’s C concordance index, which estimates the probability of concordance between observed and predicted responses, was calculated for the fully adjusted model [17]. Results are reported as hazard ratio (HR) with their respective 95% CI. The reference groups for the analyses were the healthiest category for HAI and the robust category for FI. The models were employed separately, without adjustments for HAI and FI, respectively, as they were utilized in distinct models. Two models with an incremental number of covariates were conducted: Model 1 was adjusted for sex, age, years of education, and place of residence, and model 2 was additionally adjusted for smoking status and AUDIT score. Additionally, we conducted a sensitivity analysis using a 1-year landmark, excluding all participants who experienced events within the first year of follow-up. This approach minimized the effect of reverse causality. Finally, a non-linear association of the continuous HAI and the FI with all-cause mortality was also investigated using penalized cubic splines fitted in Cox proportional hazard models. The penalized spline is a variation of the basis spline, which is less sensitive to known numbers and placements than restricted cubic splines [18]. For these analyses, variables were *z*-standardized to allow comparison among the scores. All statistical analyses were conducted using STATA V18 software (StataCorp; College Station, TX) and R 4.3.1.

## Results

The baseline characteristics of the participants stratified by HAI and FI are shown in Table 1. Overall, the HAI mean score was 4.06 (1.8) and 0.24 (0.13) for the FI. Less healthy participants (higher percentages) for both the HAI and the FI were more likely to be older, women, with less years of formal education, living in rural zones of the country, current smokers, and with a low risk of alcohol consumption (Table 1).

**Table 1:** Sociodemographic characteristics of the population in study by healthy aging index and frailty index (*n* = 847)

	Healthy aging index (*N* = 847)				Frailty index (*N* = 847)			
	Continuous (mean, SD)	0 to 3 healthiest (%)	4 to 6 intermediate (%)	7 to 10 less healthy (%)	Continuous (mean, SD)	Robust < 0.12 (%)	Pre-frail > 0.12 to 0.24 (%)	Frail > 0.24 (%)
Total	4.06 (1.8)	40.3 (37.3–43.3)	48.5 (45.5–51.6)	11.1 (9.3–13.2)	0.24 (0.13)	13.6 (11.5–15.9)	41.4 (38.4–44.5)	44.9 (41.9–48.1)
Baseline age (years), mean (SD)		67.1 (6.0)	71.4 (7.5)	76.5 (7.9)		68.1 (6.6)	69.7 (6.9)	71.6 (8.4)
Sex, (%)								
Women	4.04 (1.8)	59.1 (54.2–63.6)	61.1 (56.7–65.2)	60.4 (51.1–68.8)	0.27 (0.13)	42.8 (39.4–46.4)	59.7 (56.7–62.6)	73.7 (70.6–76.6)
Men	4.09 (1.8)	40.9 (36.3–45.7)	38.9 (34.7–43.2)	39.6 (31.1–48.8)	0.21 (0.12)	57.2 (53.5–60.5)	40.3 (37.3–43.2)	26.3 (23.4–29.3)
Educational level, (%)								
Low < 8 years	4.28 (1.8)	63.5 (58.8–68.1)	74.3 (70.3–77.9)	90.3 (83.4–94.5)	0.26 (0.13)	32.4 (29.1–35.7)	48.1 (45.1–51.1)	61.1 (58.6–65.2)
Middle 8–12 years	3.54 (1.6)	25.9 (21.9–30.3)	20.1 (16.8–23.8)	7.9 (4.1–14.5)	0.22 (0.12)	44.7 (41.2–48.3)	35.8 (32.9–38.7)	29.6 (26.5–32.7)
High > 12 years	3.28 (1.4)	10.6 (7.9–13.8)	5.6 (3.8–7.9)	1.8 (0.4–6.7)	0.21 (0.11)	22.9 (20.1–25.9)	16.1 (14.1–18.5)	8.3 (6.7–10.5)
Place of residence								
Urban	4.03 (1.7)	82.7 (78.7–85.9)	81.4 (77.8–84.5)	78.4 (70.1–85.1)	0.25 (0.13)	87.3 (84.6–89.4)	83.1 (80.6–85.2)	81.9 (79.2–84.4)
Rural	4.17 (1.8)	17.3 (14.1–21.3)	18.6 (15.4–22.2)	21.6 (14.9–29.9)	0.26 (0.12)	12.7 (10.6–15.3)	16.9 (14.8–19.4)	18.1 (15.5–20.7)
Smoking (%)								
Never	4.17 (1.8)	48.3 (43.6–53.1)	55.1 (50.7–59.4)	57.8 (48.6–66.4)	0.25 (0.12)	40.1 (36.6–43.5)	42.3 (39.2–45.3)	43.6 (40.2–46.9)
Previous	4.03 (1.7)	32.8 (28.5–37.5)	29.3 (25.4–33.7)	34.5 (26.4–43.6)	0.26 (0.13)	24.8 (21.9–27.9)	28.2 (25.5–31.1)	31.5 (28.4–34.7)
Current	3.76 (1.7)	18.9 (15.3–22.8)	15.6 (12.7–19.1)	7.7 (4.1–14.2)	0.22 (0.12)	35.1 (31.8–38.6)	29.5 (26.8–32.3)	24.9 (22.1–27.9)
AUDIT score (%)								
Low risk	4.06 (1.7)	94.1 (91.3–95.9)	94.2 (91.8–95.9)	96.6 (91.1–98.7)	0.25 (0.12)	90.2 (87.8–92.1)	92.9 (91.2–94.3)	93.9 (92.1–95.3)
Moderate risk	4.02 (1.7)	4.5 (2.9–6.9)	4.7 (3.1–6.9)	2.6 (0.8–7.7)	0.19 (0.09)	8.3 (6.5–10.4)	5.7 (4.4–7.3)	3.9 (2.8–5.4)
High risk	4 (1.5)	1.4 (0.4–2.8)	1.1 (0.4–2.8)	0.8 (0.4–2.3)	0.23 (0.12)	1.5 (0.5–2.1)	1.4 (0.6–1.8)	2.2 (1.1–2.5)

Continuous variables are expressed as mean and standard deviation (SD). Categorical variables as percentages with their 95% confidence intervals (95% CI)

**Fig. 1:**
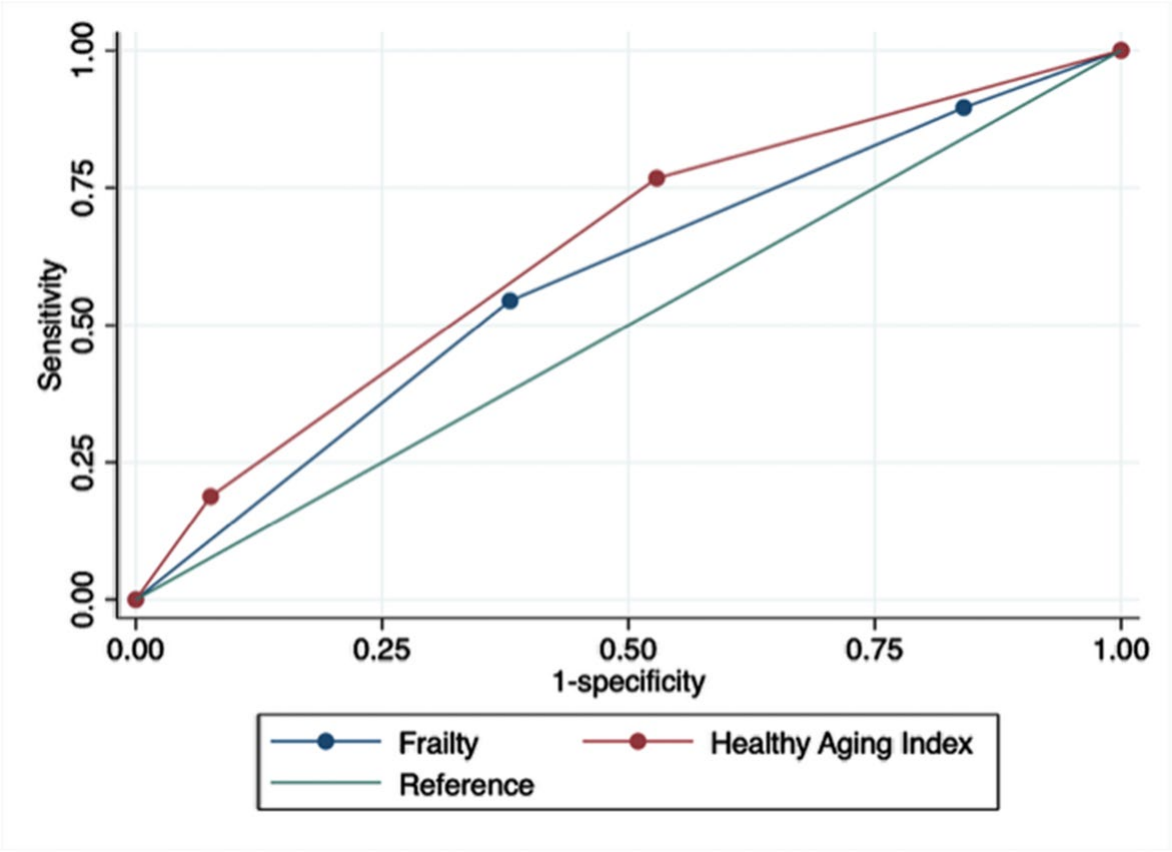
ROC curves for both the HAI and the FI

The ROC curves to assess the predictive ability for mortality for each instrument are shown in Fig. 1. While for both indexes the AUCs were low, the AUC for mortality of the HAI was higher than for the FI (0.640, [95%CI 0.601 to 0.679] vs. [0.586, 95%CI 0.545 to 0.627], respectively). Once the cut-offs were applied (Table 2), a higher prevalence of less healthy participants was observed for the FI than for the HAI (FI > 0.24 was 31.60% vs. 11.13% for those with HAI between 7 and 10 points). Moreover, higher sensitivity to detect pre-frail and frail individuals (89.61% and 57.14%) was found for the FI than the HAI to detect people categorized as intermediate and less healthy (78.74 and 20.87%). Conversely, superior sensitivity showed the HAI than the FI for the same aforementioned categories (Table 2).

**Table 2:** Prevalence, sensitivity, and specificity for different cut-offs of HAI and frailty index for all-cause mortality

	Cut-offs	Prevalence	Sensitivity	Specificity
Frailty index	> 0.12 to 0.24	39.42%	89.61%	31.4%
	> 0.24	31.60%	57.14%	71.73%
Healthy aging index	4 to 6	48.56%	78.74%	46.45%
	7 to 10	11.13%	20.87%	92.01%

**Fig. 2:**
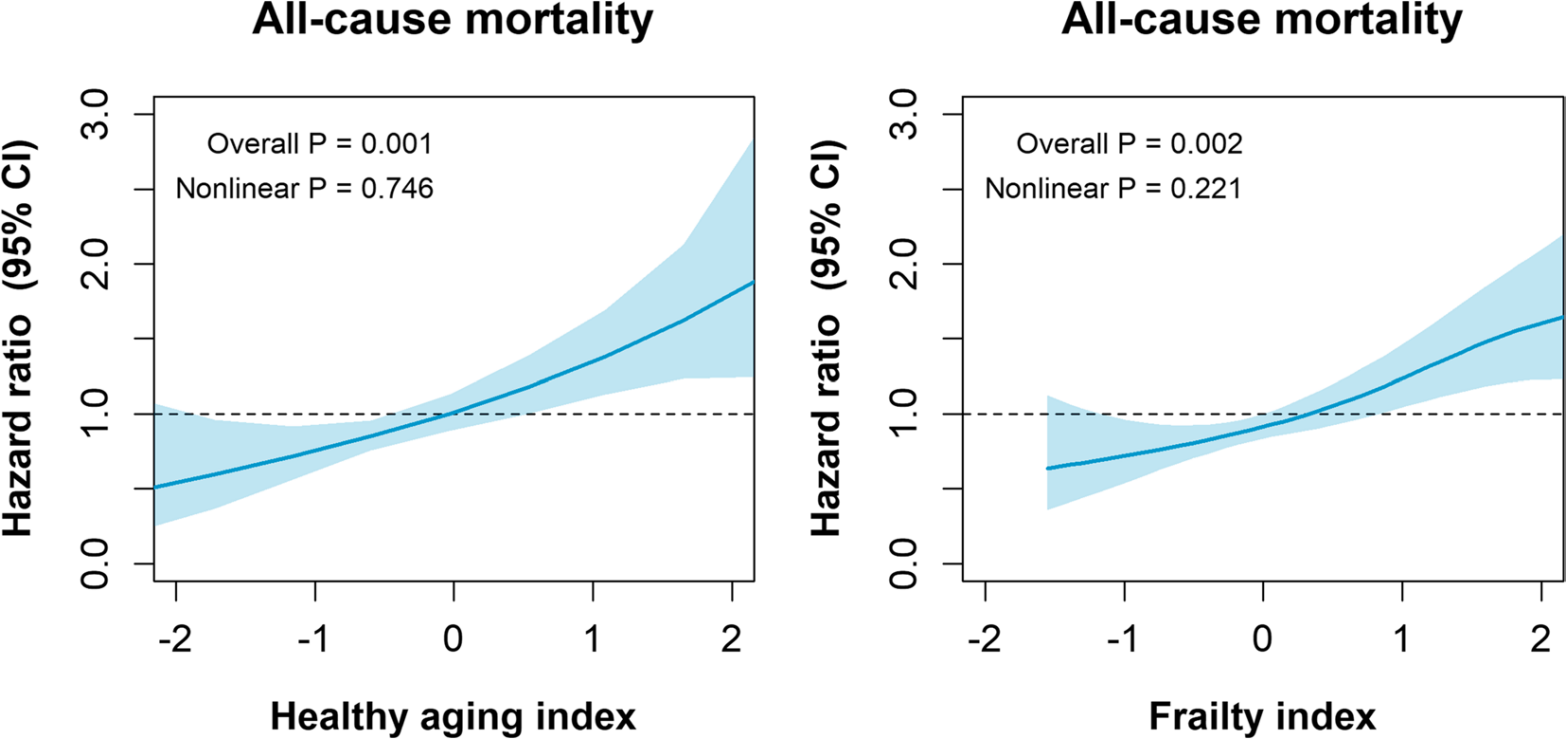
Associations between the *z*-standardized continuous healthy aging index, frailty index, and all-cause mortality in Chilean adults. Analyses are presented as HR and their 95% CI. All models were adjusted for sex, age, years of education, place of residence, and smoking and alcohol consumption

Over a median follow-up of 10.9 years (interquartile range 10.1 to 11 years), 264 (31.1%) participants died. The proportions of alive and dead people based on FI and HAI are shown in Supplementary information 2. Non-linear associations between both the HAI and the FI and all-cause mortality are presented in Fig. 2. Overall, a higher HAI and FI score was associated with a higher mortality risk (overall *p* < 0.05). No evidence of non-linearity was observed in the spline (*p* = 0.746 for the HAI and *p* = 0.221 for the FI). After adjustment for confounders (sex, age, years of education, place of residence, smoking status, and consumption of alcohol), the HR for HAI was 1.16 (95% CI 1.08 to 1.26) compared to 2.63 (95% CI 1.76 to 3.51) for the FI (Table 3). When the cut-offs were applied and after full adjustment (model 2), people in the less healthy and frail categories showed higher mortality risk for any cause than those in the healthier categories (Table 3). The risk for frail people compared to robust was 2.39 (95% CI 1.60 to 3.56) and for people with 7 to 10 points in the HAI was 2.22 (95% CI 1.55 to 3.20) compared to those with 0 to 3 points. (Table 3). Moreover, a better predictive value of mortality was observed for the FI compared to the HAI (C-index of 0.831 vs. 0.742).

**Table 3:** Association between both the FI and HAI and all-cause mortality

	Frailty index (*N* = 847)				Healthy index score (*N* = 847)			
		HR (95% CI)	HR (95% CI)	HR (95% CI)		HR (95% CI)	HR (95% CI)	HR (95% CI)
	Continuous	< 0.12	> 0.12 to 0.24	> 0.24	Continuous	0 to 3	4 to 6	7 to 10
Model 1	1.17 (1.05–1.29)	1.00 (Ref.)	1.46 (0.93–2.29)	2.30 (1.47–3.60)	2.75 (1.85–3.71)	1.00 (Ref.)	1.73 (1.30–2.29)	2.20 (1.53–3.16)
Model 2	1.16 (1.08–1.26)	1.00 (Ref.)	1.33 (0.89–2.00)	2.39 (1.60–3.56)	2.63 (1.76–3.51)	1.00 (Ref.)	1.70 (1.29–2.26)	2.22 (1.55–3.20)
1-year landmark	1.15 (1.03–1.31)	1.00 (Ref.)	1.24 (0.82–1.89)	2.19 (1.46–3.29)	2.59 (1.45–3.11)	1.00 (Ref.)	1.70 (1.23–2.44)	2.29 (1.49–3.52)

Model 1: adjusted for sex, age, years of education, and place of residence. Model 2: additionally smoking status and alcohol consumption. Model 2 is used for the 1-year landmark analysis. Analyses are presented as HR and their 95% CI

*HR*, hazard ratio; *CI*, confidence intervals

## Discussion

Using a sample of older Chileans, we developed and assessed a modified version of the HAI to compare its predictive ability with the Chilean version of the FI. Our study identified that both the FI and HAI were valid and reliable predictors for all-cause mortality in the Chilean population. The HAI exhibited a higher predicted ability for mortality (AUC) than the FI, while the FI demonstrated better sensitivity. However, when the C-index was calculated, the FI showed better prediction. Additionally, the FI showed higher HRs for mortality compared to the HAI in the adjusted Cox regression models. Furthermore, we observed higher scores on both the FI and the HAI among older individuals, women, and individuals with lower levels of education (8 years or less of formal education).

Our findings are consistent with previous studies. Wu et al. [10] reported that a modified version of the HAI (including systolic blood pressure, Digit Symbol Substitution Test, cystatin C, glucose, and respiratory problems) was linked to all-cause and cardiovascular mortality in older Americans, with a HR of 1.19 (95% CI 1.11 to 1.27) and 1.23 (95% CI 1.11 to 1.35), respectively. This study also revealed a higher prevalence of elevated HAI scores among individuals with higher education levels and older age. Additionally, Huang et al. [11], using a similar version of the HAI, reported its association with an increased risk of major cardiovascular events. Each point increase in the HAI was associated with a 44% higher risk of major cardiac events (HR 1.44 (95% CI 1.40 to 1.49)), 44% higher risk of major coronary events (HR, 1.44 (95% CI 1.40 to 1.48)), and 36% higher risk of ischemic heart disease (HR 1.36 (95% CI 1.33 to 1.39)). Although we lacked data on specific cardiovascular events such as myocardial infarction or stroke, it is well established that these conditions are connected to higher mortality rates, particularly among older individuals like those in our study [19–21]. Notably, we could not find information regarding the development and validation of the HAI in other Latin American countries.

Two previous studies examined the changes in the HAI over time. Connell et al. [22] showed that HAI tends to increase with advancing age after a 9-year follow-up period, and this increase was linked to higher mortality rates. Similarly, Dieteren et al. [23] reported that the trajectories of HAI vary between women and men and are influenced by factors such as age, educational level, physical activity, and body mass index. These reports also underscore the significant role of sociodemographic and lifestyle factors in the aging process, some of which are associated with higher mortality risk, particularly among older populations.

To the best of our knowledge, this is the first study to compare the predictive ability of mortality, specificity, and sensitivity of the two health indices in Latin America. We have shown that both the FI and the HAI were reliable predictors of mortality in older individuals. It is important to acknowledge that the FI and the HAI capture distinct aspects of aging and health. The FI focuses on identifying frail individuals and assessing associated risks, while the HAI takes a more comprehensive approach by considering positive dimensions of health. Both indices offer valuable tools for evaluating and monitoring the health of older populations, although their application may vary depending on the specific research or clinical objectives. Previous research has indicated that HAI can predict the decline of various age-related functions, including slow gait speed, multimorbidity, and disability, which are essential components of frailty [13, 24, 25].

This research possesses several notable strengths. It is the first study to develop and validate a modified version of the HAI in Chile and Latin America and to compare its properties with the Chilean version of the FI. Additionally, we characterized the distribution of this HAI version using a nationally representative sample of older individuals, ensuring adequate representation of different regions of the country and sociodemographic characteristics.

However, there are some limitations to this study. Firstly, we relied on self-reported respiratory problems as a surrogate for forced vital capacity due to the lack of objective pulmonary function measurements in the CHNS. Secondly, all five components of the HAI were measured only once, which may lead to potential misclassification as these measures can vary over time. Thirdly, the observational nature of the data restricts our ability to infer causality from the results. Finally, despite adjusting for important confounders, there remains the possibility of unmeasured confounders, such as muscle measurements, influencing the outcomes.

In conclusion, both the FI and the HAI were valuable tools for understanding frailty and healthy aging, respectively. These indices enable a comprehensive assessment of deficits and diverse aspects of health, thereby providing valuable insights into older adults’ well-being and health status. Integrating these indices into research and clinical practice can significantly enhance our capacity to identify at-risk individuals, promote healthy aging, and guide interventions to improve health outcomes in aging populations.

### Supplementary Information

Below is the link to the electronic supplementary material.

Supplementary file1 (DOCX 18 KB)

## Data Availability

These data were derived from the following resources available in the public domain: website of the Epidemiology Department of the Ministry of Health, Chile: http://www.epi.minsal.cl/encuesta-ens/.
