# Supplementary material for: Predictive ability of both the healthy aging index and the frailty index for all-cause mortality
Source: GeroScience. 2024 Feb 22;46(3):3471–9. doi: 10.1007/s11357-024-01097-0 (PMC11009179; doi:10.1007/s11357-024-01097-0)
Supplement: Supplementary file 1 — Supplementary file1 (DOCX 18 KB) [file 11357_2024_1097_MOESM1_ESM.docx]

| Score for group | **Lung function**  (Expectoration, cough, sibilance, and difficulty in breathing) | **Systolic blood pressure**  (mm/Hg) | **Fasting glucose**  (mg/dl) | **Cognitive status**  **(MMSE)** | **Glomerular filtration rate** |
| --- | --- | --- | --- | --- | --- |
| 0 = healthiest | O symptoms | <124 | <100 | 17 to max | >90 |
| 1 = intermediate | 1 or 2 symptoms | 124-139 | 101-125 | 14 to 16 | 60 to 89 |
| 2 = less healthy | 3 or more symptoms | >139 | >125 | <14 | <60 |

**PREDICTIVE ABILITY OF BOTH THE HEALTHY AGING INDEX AND THE FRAILTY INDEX FOR ALL-CAUSE MORTALITY**

**Supplement 1. Cut-off used to create the Healthy Aging Index.**

- MMSE: Mini Mental State Examination.
- Glomerular filtration rate: this was calculated using the plasma value of creatinine through the Cockcroft formula.

**Supplement 2. Mortality for Frailty Index and Healthy Aging Index**

|  | **Cutoffs** | **Alive**  **n (%)** | **Dead**  **n (%)** | **p-value** |
| --- | --- | --- | --- | --- |
| **Frailty Index** | **≤0.12** | 59 (10.1) | 15 (5.7) | <0.001 |
|  | **>0.12 to 0.24** | 296 (50.7) | 106 (40.1) |  |
|  | **>0.24** | 228 (39.2) | 143 (54.2) |  |
| **Healthy Aging Index** | **0 to 3** | 288 (49.4) | 63 (23.9) | <0.001 |
|  | **4 to 6** | 255 (43.7) | 154 (58.3) |  |
|  | **7 to 10** | 40 (6.9) | 47 (17.8) |  |
